# Cardiovascular risk assessment for patients with serious mental illnesses: An internal review

**DOI:** 10.5339/qmj.2021.32

**Published:** 2021-09-06

**Authors:** Mahmoud Alshawwaf, John Delson Molina, Yassin Eltotrki, Mohamed Adil Shah Khoodoruth, Majid Alabdulla

**Affiliations:** ^1^Department of Psychiatry, Hamad Medical Corporation, Doha, Qatar E-mail: mah1986@yahoo.com; ^2^College of Medicine, Qatar University, Doha, Qatar

## Abstract

Cardiovascular diseases (CVD) are the leading cause of excess premature mortality among patients with serious mental illness (SMI), mainly because of higher cardiovascular risk and metabolic syndrome compared to the general population.^1,2^ A pertinent contributing factor is second-generation antipsychotics, which further negatively impact the cardiovascular risk burden, amounting to a significant clinical and public health challenge among patients with SMI.^3^

Qatar has a high metabolic syndrome prevalence of 26%, and the blood pressure of patients with SMI receiving antipsychotics is significantly higher.^4^ In 2019, the Pharmacy Department of Mental Health Services at Hamad Medical Corporation (HMC) in Doha, Qatar flagged four moderately and one mildly severe cases of adverse drug reaction secondary to antipsychotics. In response to above mentioned incidents, this quality improvement (QI) project was conducted in an acute inpatient male ward from November 2019 to June 2020 in the Psychiatry Hospital of Hamad Medical Corporation to implement a cardiovascular risk assessment for inpatients with SMI.

The atherosclerotic cardiovascular disease (ASCVD) risk estimator was used to estimate the 10-year risk of CVD, and inpatients were categorized into low-risk ( < 5%), borderline risk (5%–7.4%), intermediate-risk (7.5%–19.9%), and high risk ( ≥ 20%).^5^ Patients with SMI above 40 years of age were included. Within 72 hours of admission, the admitting inpatient nurse filled a cardiovascular risk assessment (CVRA) questionnaire, including basic demographics, past and present cardiology and smoking history, laboratory test results such as lipid panel, and renal function tests. This study used an in-depth, semi-structured face-to-face interview as a primary data collection technique. An interview guide was developed to address the purpose. At the end of each assessment, the QI nurse approached the participants again to educate them about the cardiovascular risk result accordingly and explained the required referrals (Cardiology and/or Smoking Cessation). The QI team member calculated the cardiovascular risk by utilizing the ASCVD plus application to determine inpatient cardiovascular risk.

Out of 26 inpatients with SMI who underwent CVRA, nine (35%) scored moderate to high risk, and were referred to Cardiology for further intervention. Among these nine patients, two (22%) were started on statin therapy, three (33%) started on aspirin, and the remaining four (44%) received lifestyle modifications advice and counseling. Ten (38%) were referred to a smoking cessation clinic for nicotine replacement therapy and counseling. Table 1 shows the clinical characteristics of patients included in this study. Three inpatient consultations to cardiology were rejected, which shows how health professionals underestimate and stigmatize effective interventions for patient with SMI. In case of comorbidities of two diseases, one of them is known overlooked and this is particularly true for mental illness.^6^ A cohort study on medical comorbidities in patients with SMIs in Qatar concluded that an evidence of individuals with SMI is less likely to receive standard levels of care for their medical comorbidities.^7^ Mental health training could help medical health professionals from other specialties improve their understanding on the impact of both psychotropic medications and mental illness in the physical health of people with SMI and address the fear and stigma. Communication improvement between professionals by meaningful discussion of objectives of referral with the patient and contacting the consultant referred to might improve coordination among the referring psychiatrist, physician referred to, and patient.^8^

Patients who scored high on (ASCVD) assessment and are asymptomatic might benefit from referral to primary health care centers for further assessment by a generalist from whom a referral to other specialty like cardiology might be more easily accepted. From the patients’ perspective, physical health education, and most importantly, cardiovascular risk assessment are now significantly invested. All patients above 40 years old with SMI will undergo a CVRA. Besides, their assigned nurse will work jointly with allied health professionals to educate them about the importance of healthy lifestyle, including healthy diet and staying fit. Tailor-made recommendations will be established, taking into consideration the cardiovascular risk status and antipsychotic medications. Close participation with the clinical pharmacist and dietician will ensure constant psychoeducation about the metabolic side effects of antipsychotic medications. Physiotherapists will identify barriers, if any, for patients to participate in physical activities offered by the ward. Simultaneously, facilitators will include one-to-one contact with staff and work on increasing awareness of the positive impact of physical activity at the departmental level. Leadership involvement is crucial to ensure joint agreement with different specialties, particularly those based in other HMC facilities, such as cardiology and smoking cessation clinics, to accept referrals when required.

## Figures and Tables

**Table 1:** Clinical characteristics of 26 inpatients with SMI

Characteristic	

Age in years (Mean and SD)	49.0 (6.9)

History of CVD (n (%))	

Yes	7 (26.9)

Unknown	10 (38.5)

Hypertensive medications (n (%))	

Yes	8 (30.8)

Diabetic (n (%))	

Yes	17 (65.4)

HbA1c (Mean and SD)	6.0 (1.3)

Smoking status (n (%))	

Smoker	13 (50)

Body mass index (n (%))	

Healthy	5 (19.2)

Overweight	11 (42.3)

Obese	10 (38.5)

Lipid panel (mean and SD)	

Total cholesterol (mg/dl)	4.7 (1.0)

HDL (mg/dl)	1.2 (0.2)

LDL (mg/dl)	2.7 (1.0)

Blood pressure ((mean and SD)	

Systolic (mmHg)	131 (11)

Diastolic (mmHg)	86 (23)

ASCVD risk classification (n (%))	

Low	17 (65)

Moderate	8 (31)

Severe	1 (4)


ASCVD: Atherosclerotic cardiovascular disease CVD: Cardiovascular disease HbA1c: Hemoglobin A1c SD: Standard deviation
